# Prognostic value of computed tomography score in patients after extracorporeal cardiopulmonary resuscitation

**DOI:** 10.1186/s13054-018-2101-2

**Published:** 2018-11-22

**Authors:** Jeong-Am Ryu, Young Hwan Lee, Chi Ryang Chung, Yang Hyun Cho, Kiick Sung, Kyeongman Jeon, Gee Young Suh, Taek Kyu Park, Joo Myung Lee, Minjung Kathy Chae, Jeong-Ho Hong, Sei Hee Lee, Hyoung Soo Kim, Jeong Hoon Yang

**Affiliations:** 1 Department of Critical Care Medicine, Samsung Medical Center, Sungkyunkwan University School of Medicine, 81 Irwon-ro, Gangnam-gu, Seoul, 06351 Republic of Korea; 2 Department of Neurosurgery, Samsung Medical Center, Sungkyunkwan University School of Medicine, Seoul, South Korea; 3 Department of Emergency Medicine, Soonchunhyang University Bucheon Hospital, Bucheon, South Korea; 4 Department of Thoracic and Cardiovascular Surgery, Samsung Medical Center, Sungkyunkwan University School of Medicine, Seoul, Republic of Korea; 5 Division of Pulmonary and Critical Care Medicine, Department of Medicine, Samsung Medical Center, Sungkyunkwan University School of Medicine, Seoul, South Korea; 6 Division of Cardiology, Department of Medicine, Samsung Medical Center, Sungkyunkwan University School of Medicine, Seoul, Republic of Korea; 7 Department of Emergency Medicine, Ajou University School of Medicine, Suwon, South Korea; 8 Department of Neurology, Keimyung University Dongsan Medical Center, Daegu, South Korea; 9 Department of Emergency Medicine, Hallym University Medical Center, Anyang, South Korea; 10 Department of Thoracic and Cardiovascular Surgery, Hallym University Medical Center, Anyang, South Korea

**Keywords:** Brain computed tomography, Cardiopulmonary resuscitation, Extracorporeal membrane oxygenation

## Abstract

**Background:**

We evaluated whether Alberta Stroke Program Early Computed Tomography Score (ASPECTS) with some modifications could be used to predict neurological outcomes in patients after extracorporeal cardiopulmonary resuscitation (ECPR).

**Methods:**

This was a retrospective, multicenter, observational study of adult unconscious patients who were evaluated by brain computed tomography (CT) within 48 hours after ECPR between May 2010 and December 2016. ASPECTS, bilateral ASPECTS (ASPECTS-b), and modified ASPECTS (mASPECTS) were assessed by ROC curves to predict neurological outcomes. The primary outcome was neurological status upon hospital discharge assessed with the Cerebral Performance Categories (CPC) scale.

**Results:**

Among 58 unconscious patients, survival to discharge was identified in 25 (43.1%) patients. Of these 25 survivors, 19 (32.8%) had good neurological outcomes (CPC score of 1 or 2). Interrater reliability of CT scores was excellent. Intraclass correlation coefficients of ASPECTS, ASPECTS-b, and mASPECTS were 0.918 (95% CI, 0.865–0.950), 0.918 (95% CI, 0.866–0.951), and 0.915 (95% CI, 0.860–0.949), respectively. The predictive performance of mASPECTS for poor neurological outcome was better than that of ASPECTS or ASPECTS-b (C-statistic for mASPECTS vs. ASPECTS, 0.922 vs. 0.812, *p* = 0.004; mASPECTS vs. ASPECTS-b, 0.922 vs. 0.818, *p* = 0.003). A cutoff of 25 for poor neurological outcome had a sensitivity of 84.6% (95% CI, 69.5–94.1%) and a specificity of 89.5% (95% CI, 66.9–98.7%) in mASPECTS.

**Conclusions:**

mASPECTS might be useful for predicting neurological outcomes in patients after ECPR.

**Electronic supplementary material:**

The online version of this article (10.1186/s13054-018-2101-2) contains supplementary material, which is available to authorized users.

## Background

Neurological outcome is an important issue in patients who survive cardiac arrest. In these survivors, several predictors of neurological outcomes, such as physical examination, several biomarkers, and electrophysiologic studies, have been reported [1, 2]. Brain imaging might also be helpful for predicting neurological outcomes after cardiac arrest [3, 4]. Acute hypoxic-ischemic injury might be manifested by loss of gray-white matter discrimination, brain swelling, or low-density lesions on brain computed tomography (CT) [5, 6]. However, these changes in brain CT are likely to be subjective and difficult to quantify [7]. The Alberta Stroke Program Early Computed Tomography Score (ASPECTS) is a widely used screening tool that provides a framework for quantifying the extent of ischemic hypodensity or hypoattenuation in the middle cerebral arterial (MCA) territory [8]. In the setting of conventional cardiopulmonary resuscitation (CPR), ASPECTS with some modifications has been found to be useful to predict neurological outcomes of post-cardiac arrest patients [9, 10]. However, whether ASPECTS might be helpful to systemically estimate neurological outcomes of survivors after extracorporeal cardiopulmonary resuscitation (ECPR) has not been reported. Therefore, the objective of this study was to investigate whether ASPECTS with some modifications could be used to predict neurological outcomes of patients after ECPR.

## Methods

### Study population

**Fig. 1:**
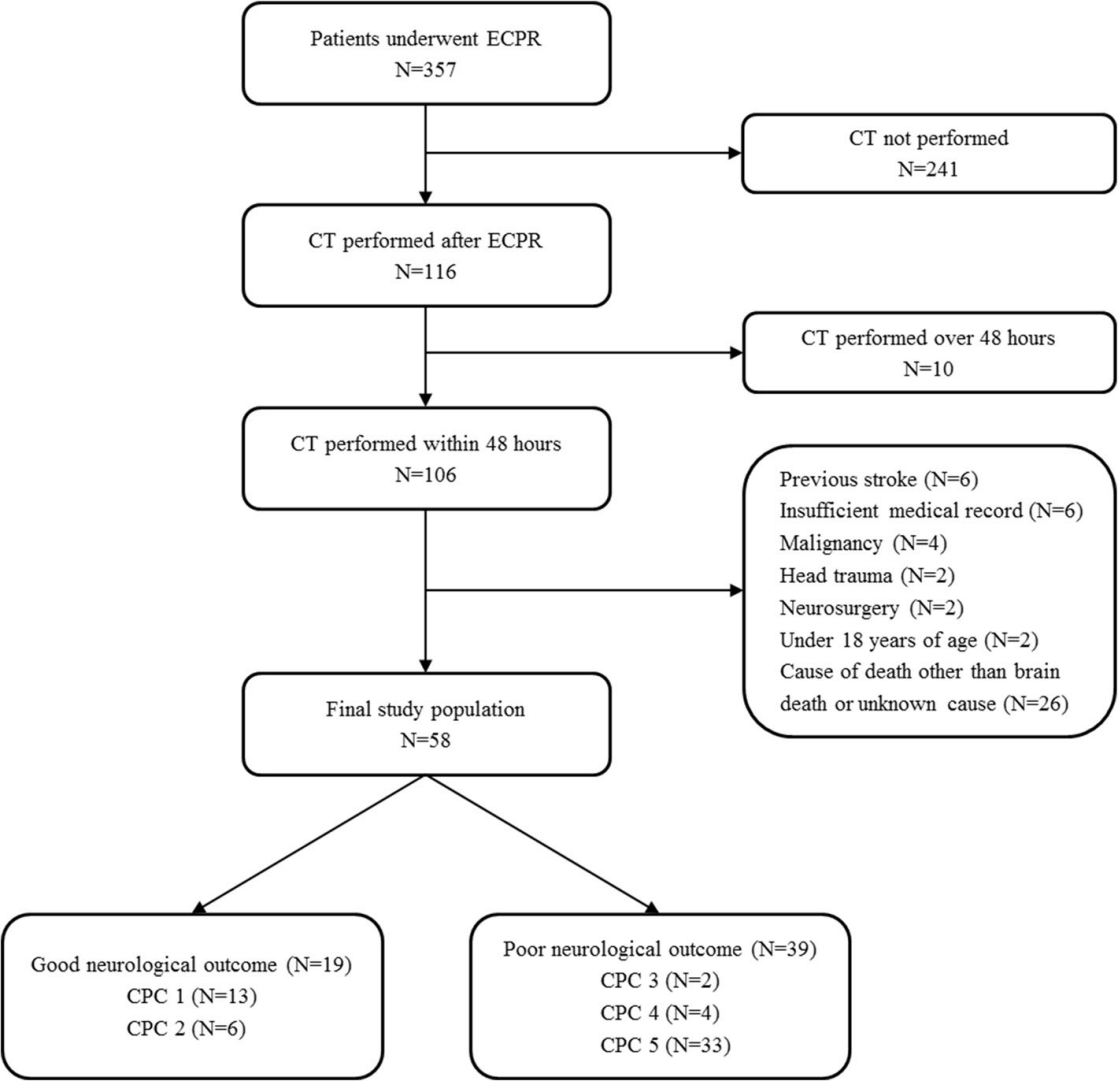
Study flowchart. *ECMO* Extracorporeal membrane oxygenation, *CPR* Cardiopulmonary resuscitation, *ECPR* Extracorporeal cardiopulmonary resuscitation, *CPC* Cerebral Performance Categories scale

This was a retrospective, multicenter, observational study of adult patients who underwent ECPR during hospitalization at Hallym University Medical Center (HUMC) and Samsung Medical Center (SMC) between May 2010 and December 2016. This study was approved by the institutional review boards of HUMC (HUMC 2015i128) and SMC (SMC 2017-11-088-002). The requirement for informed consent was waived owing to the study’s retrospective nature. Clinical and laboratory data were collected by a trained study coordinator using a standardized case report form. We included patients who underwent ECPR during the study period. Those who were unconscious (a score < 9 on the Glasgow Coma Scale) [7] upon admission to the hospital after cardiac arrest and those who had a brain CT scan within 48 hours after ECPR were selected. Of these patients, we excluded patients who were under 18 years of age; those who had malignancy whose expected lifespan was less than 1 year; those who had insufficient medical records; and those who had a history of head trauma, neurosurgery, or chronic neurological abnormality upon intensive care unit (ICU) admission. In addition, we excluded patients who had low ASPECTS and were suspected to have brain lesions due to factors other than hypoxic ischemic encephalopathy. We excluded patients for whom we could not define neurological status because of continuous sedation or death of unknown causes or causes other than brain death. Finally, a total of 58 patients with cardiac arrest who were rescued by venoarterial extracorporeal membrane oxygenation (ECMO) were analyzed in this study (Fig. 1).

### Definitions and outcomes

**Fig. 2:**
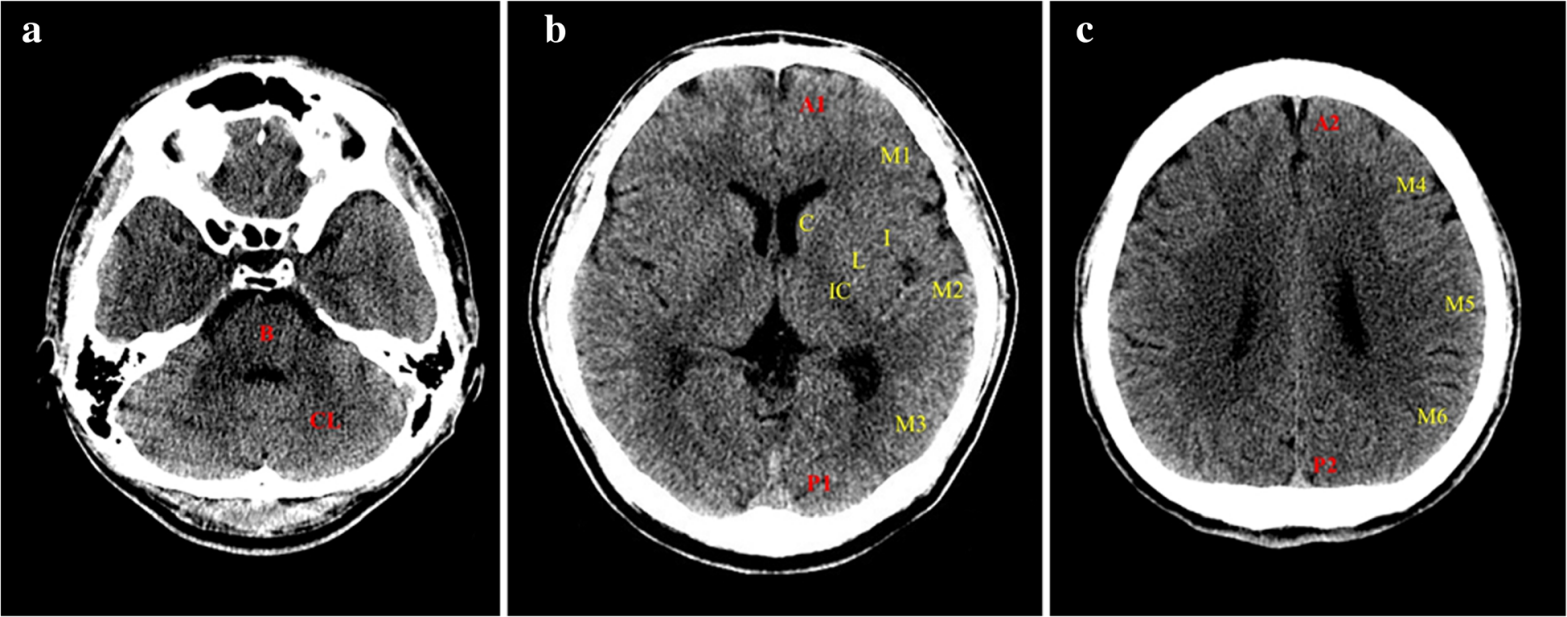
Axial computed tomographic images at three levels (**a**, **b**, **c**). Modified Alberta Stroke Program Early Computed Tomography Score (ASPECTS) was used for analysis of images. The score was used to interpret the whole brain. It was not limited to specific slices. *See* Table 1 for further details. Bilateral yellow regions indicate ASPECTS-b (extended to both sides from the original ASPECTS protocol). Combined bilateral regions of yellow and red indicate modified ASPECTS. *A1* Anterior cerebral artery (ACA) lower, *A2* ACA upper, *ASPECTS-b* Bilateral ASPECTS, *B* Brainstem, *C* Caudate, *CL* Cerebellum, *I* Insula, *IC* Internal capsule, *L* Lentiform nucleus, *M1–M6* Middle cerebral artery territories, *mASPECTS* Modified ASPECTS, *P1* Posterior cerebral artery (PCA) lower, *P2* PCA upper

In this study, ECPR was defined as both successful venoarterial ECMO implantation and pump-on with cardiac massage during index procedure in patients with cardiac arrest. When a return of spontaneous circulation (ROSC) occurred during ECMO cannulation, practitioners typically did not remove the cannula or stop the ECMO pump-on process [11, 12]. ECMO pump-on was defined by stopping chest compressions following successful ECMO implantation and activation. ECMO flow was then gradually increased until the patient was hemodynamically stable. The resuscitation procedure was performed in the same way as described in our previous study [7, 13]. Arrest to ECMO pump-on time was defined as the time from cardiac arrest to the time at which the ECMO pump was turned on. Targeted temperature management was performed with surface cooling devices. We used a commercial temperature regulation system consisting of a hydrogel pad (Arctic Sun®; Medivance Corp., Louisville, CO, USA) or a cooling blanket. Surface cooling and the targeted temperature were determined by each intensivist in the ICU according to the therapeutic hypothermia protocol [14]. The primary outcome was neurological status upon hospital discharge assessed with the Glasgow-Pittsburgh Cerebral Performance Categories (CPC) scale (1 to 5) [15]. CPC scores of 1 and 2 were classified as good neurological outcomes. CPC scores of 3, 4, and 5 were considered as poor neurological outcomes. We thoroughly reviewed medical records. Patients were graded on the CPC scale by two independent neurologists. Brain death was confirmed by neurologists. Brain death was diagnosed on the basis of absence of brainstem reflex by neurologic examination, electroencephalography (EEG), and apnea test. Sometimes, transcranial Doppler or brain CT angiography was needed to diagnose brain death when apnea test was impossible due to extracorporeal circulation. Prognostication upon electrocerebral inactivity on EEG and apnea led to termination of therapy or consideration of organ transplant. All recorded brain CT scans were taken within 48 hours after ROSC in this study. After successful ECPR, for patients who had a rapid recovery of mental status and neurological deficits, brain CT was not performed. Otherwise, brain CT was performed to determine whether control of increased intracranial pressure was needed. Brain CT was also used to exclude intracranial hemorrhage before therapeutic hypothermia by the intensivist. For all CT studies, 64-channel scanners (at HUMC, SOMATOM Sensation; Siemens, Erlangen, Germany; at SMC, Light Speed VCT; GE Healthcare, Milwaukee, WI, USA) with a 5-mm slice width were used. Brain CT images were reviewed by two independent neurologists. Investigators who were blinded to clinical information opened these CT scans for each patient using commercial image-viewing software (at HUMC, PiView STAR; INFINITT Healthcare, Seoul, South Korea; at SMC, Centricity RA1000 PACS Viewer; GE Healthcare, Milwaukee, WI, USA). To evaluate the extent of hypoxic-ischemic insult quantitatively, newly modified CT scores were used that are based on the original ASPECTS protocol [8]. The original ASPECTS was used to predict neurological outcomes in this study. Of original ASPECTSs for both hemispheres, the smaller value was used for analysis. To evaluate brain injuries in both hemispheres, bilateral ASPECTS (ASPECTS-b) was extended to both sides based on the original ASPECTS protocol [10]. In addition, modified ASPECTS (mASPECTS) was used in this study. Similar to ASPECTS-b, each MCA territory was scored with 0–10 points. Anterior cerebral artery (ACA) and posterior cerebral artery (PCA) territories had 2 points corresponding to upper and lower levels of each territory. The brainstem and each cerebellar hemisphere had 1 point (Table 1, Fig. 2) [6]. Whenever there were early ischemic findings on CT images such as parenchymal hypoattenuation, cortical swelling with sulcal effacement, and loss of gray-white matter differentiation from each area, new CT scores were calculated by subtracting 1 point from the maximum of 20 (ASPECTS-b) or 31 (mASPECTS) [6, 10]. A brain with diffuse infarction that involved all areas was scored zero.

**Table 1:** Scoring system of modified ASPECTS

	Side		Scoring
Vascular territories	Right	Left	
ACA lower (A1)	1	1	2
ACA upper (A2)	1	1	2
MCA (ASPECTS and ASPECTS-b)			20
MCA as per ASPECTS (C, caudate; I, insula; L, lentiform nucleus; IC, internal capsule; MCA, M1-M6)	0–10	0–10	
PCA lower (P1)	1	1	2
PCA upper (P2)	1	1	2
Cerebellum (CL)	1	1	2
Brainstem (B)	1		1
Total score			31

*Abbreviations: ACA* Anterior cerebral artery, *ASPECTS* Alberta Stroke Program Early CT Score, *MCA* Middle cerebral artery, *PCA* Posterior cerebral artery

### Resuscitation procedure

CPR was led by the hospital’s CPR team, and all data related to the CPR scene were recorded by a bedside nurse according to the Utstein-style guidelines [16]. The on-call ECMO team leader was called when CPR was performed for longer than 10 minutes or in the event of unstable vital signs or recurrent cardiac arrest. The ECMO team leader along with the CPR leader assessed the patient and decided whether to institute ECPR. ECPR was performed when a witnessed arrest was confirmed, the arrest persisted despite at least 10 minutes of conventional CPR, and the underlying cause of the arrest was considered reversible [17]. Cases in which ECPR was deferred included a short life expectancy (< 6 months), terminal malignancy, an unwitnessed collapse, limited physical activity, an unprotected airway, or CPR undertaken for longer than 60 minutes at the time of initial contact. Age alone did not constitute a contraindication for ECPR [17]. The ECMO team at our institution consists of cardiologists, cardiovascular surgeons, intensivists, specialized nurses, and perfusionists. Either the Capiox Emergency Bypass System (Terumo, Tokyo, Japan) or the Prolonged Life Support System (Maquet Cardiopulmonary, Hirrlingen, Germany) was used in all cases. A crystalloid solution such as normal saline or balanced solution was used for priming; no patient in this study underwent blood-primed ECMO. A percutaneous vascular approach was tried initially in all cases using the Seldinger technique; if percutaneous cannulation failed, a surgical cut-down exposure was performed [17]. The femoral vessels were the most common sites of vascular access, and 14- to 17-French arterial cannulas and 20- to 24-French venous cannulas were placed [12]. Cardiac compression was stopped once ECMO initiation was deemed successful. Anticoagulation was accomplished with a bolus injection of unfractionated heparin followed by a continuous intravenous heparin infusion to maintain an activated clotting time between 150 and 180 seconds. The initial number of revolutions per minute of the ECMO device was adjusted to achieve an ideal cardiac index greater than 2.2 L/min/m^2^ of body surface area, central mixed venous oxygen saturation above 70%, and a mean arterial pressure above 65 mmHg [12]. Blood pressure was monitored continuously through an arterial catheter, and an artery in the right arm was used for arterial blood gas analysis to estimate cerebral oxygenation. After ECMO was established, the necessary steps were taken to treat the underlying cause of cardiac arrest, including percutaneous coronary intervention, coronary artery bypass grafting, heart transplant, and noncardiopulmonary surgery [12].

### Statistical analyses

All data are presented as median and interquartile range (IQR) for continuous variables and number (percent) for categorical variables. Data were compared using the Mann-Whitney *U* test for continuous variables and the chi-square test or Fisher’s exact test for categorical variables. The predictive performance of each brain CT marker was assessed using AUC of the ROC curve for sensitivity vs. 1 − specificity. AUCs were compared using the nonparametric approach published by DeLong et al. [18] for two correlated AUCs. The optimal cut-off value of each brain CT marker for predicting poor neurological outcome was obtained by the ROC curve and Youden index [19, 20]. Intraclass correlation coefficient (ICC) was used to analyze interrater reliability of ASPECTS-b and mASPECTS [21]. ICC was estimated using a single-measurement absolute-agreement and two-way mixed-effects model [21]. All tests were two-sided, and *p* values < 0.05 were considered statistically significant. Data were analyzed using IBM SPSS statistics version 20 software (IBM, Armonk, NY, USA).

## Results

### Baseline characteristics and clinical outcomes

The median age of patients was 52 years (IQR, 37–58 years). Of 58 patients, 44 (75.9%) were males. A cardiac cause of arrest was verified in 49 (84.5%) patients. Thirty-one (53.4%) patients experienced cardiac arrest in the hospital, and 27 (46.6%) patients had cardiac arrest in an out-of-hospital setting. The median time from arrest to ECMO pump-on was 47.5 minutes (IQR, 29.0–63.0 minutes). Targeted temperature management was performed in 40 (69.0%) patients using surface cooling devices. Only one patient had ROSC during cannulation. Baseline characteristics of ECPR patients are presented in Table 2. Characteristics of cardiac arrest are shown in Table 3. There were no significant differences in baseline or arrest-related characteristics between the two neurological outcome groups. Successful ECMO weaning was achieved in 35 (60.3%) patients. Survival to discharge was identified in 25 (43.1%) patients. Of these 25 survivors, 19 (32.8%) had good neurological outcomes (CPC score of 1 or 2) (Fig. 1).

**Table 2:** Baseline characteristics of patients

	Good neurological outcome (*n* = 19)	Poor neurological outcome (*n* = 39)	*p* Value
Age, yr, median (IQR)	50.0 (32.0–54.5)	54.0 (45.0–61.0)	0.136
Gender, male, *n* (%)	15 (78.9)	29 (74.4)	0.955
Body mass index, kg/m^2^	23.1 (21.6–25.8)	25.5 (22.4–28.3)	0.064
Medical history, *n* (%)			
Diabetes mellitus	3 (15.8)	15 (38.5)	0.147
Hypertension	6 (31.6)	18 (46.2)	0.439
Malignancy	1 (5.3)	5 (12.8)	0.669
Dyslipidemia	1 (5.3)	6 (15.4)	0.496
Current smoker	6 (31.6)	14 (35.9)	0.976
Previous myocardial infarction	0 (0)	6 (15.4)	0.178
Targeted temperature management, *n* (%)			0.928
Arctic Sun®	11 (57.9)	24 (61.5)	
Cooling pad	2 (10.5)	3 (7.7)	
Initial Glasgow Coma Scale score, median (IQR)	3.0 (3.0–3.0)	3.0 (3.0–3.0)	0.208
Laboratory data on admission			
Initial lactate, mmol/L	12.4 (10.1–15.1)	8.8 (7.3–13.3)	0.071
Hemoglobin before ECMO, g/dl	15.0 (12.6–15.5)	13.2 (11.3–14.8)	0.099
Hemoglobin after ECMO, g/dl	11.1 (9.8–12.4)	12.1 (9.7–13.1)	0.449
Total bilirubin, mg/dl	0.7 (0.3–0.9)	0.8 (0.5–1.1)	0.210
Blood urea nitrogen, mg/dl	15.0 (10.6–18.0)	15.5 (13.0–23.5)	0.223
Creatinine, mg/dl	1.1 (1.0–1.3)	1.2 (1.0–1.7)	0.292
Bicarbonate, IU/L	11.7 (10.3–17.9)	17.7 (13.2–20.9)	0.121

*IQR* Interquartile range, *ECMO* Extracorporeal membrane oxygenation

**Table 3:** Characteristics of patients with cardiac arrest and procedure

	Good neurological outcome (*n* = 19)	Poor neurological outcome (*n* = 39)	*p* Value
Type of cardiac arrest, *n* (%)			0.451
Out-of-hospital cardiac arrest	7 (36.8)	20 (51.3)	
In-hospital cardiac arrest	12 (63.2)	19 (48.7)	
Bystander-witnessed cardiac arrest, *n* (%)	19 (100.0)	35 (89.7)	0.371
Bystander-performed CPR, *n* (%)	15 (78.9)	30 (76.9)	0.999
First monitored rhythm, *n* (%)			0.474
Asystole	2 (11.8)	10 (26.3)	
Pulseless electrical activity	6 (35.3)	12 (31.6)	
Shockable rhythm (VT or VF)	9 (52.9)	16 (42.1)	
Defibrillation, *n* (%)	11 (61.1)	18 (47.4)	0.500
Arrest to ECMO pump-on time, min, median (IQR)	47.0 (19.0–55.0)	48.0 (34.5–65.0)	0.359
Location of ECMO insertion, *n* (%)			0.594
Intensive care unit	3 (15.8)	7 (17.9)	
Catheterization room	7 (36.8)	19 (48.7)	
Emergency room	9 (47.4)	12 (30.8)	
Other	0 (0)	1 (2.6)	
Cardiac cause of arrest, *n* (%)			0.156
Ischemic	6 (37.5)	21 (63.6)	
Nonischemic	10 (62.5)	12 (36.4)	

*Abbreviations: CPR* Cardiopulmonary resuscitation, *VT* Ventricular tachycardia, *VF* Ventricular fibrillation, *ECMO* Extracorporeal membrane oxygenation, *IQR* Interquartile range

### Brain CT findings

**Fig. 3:**
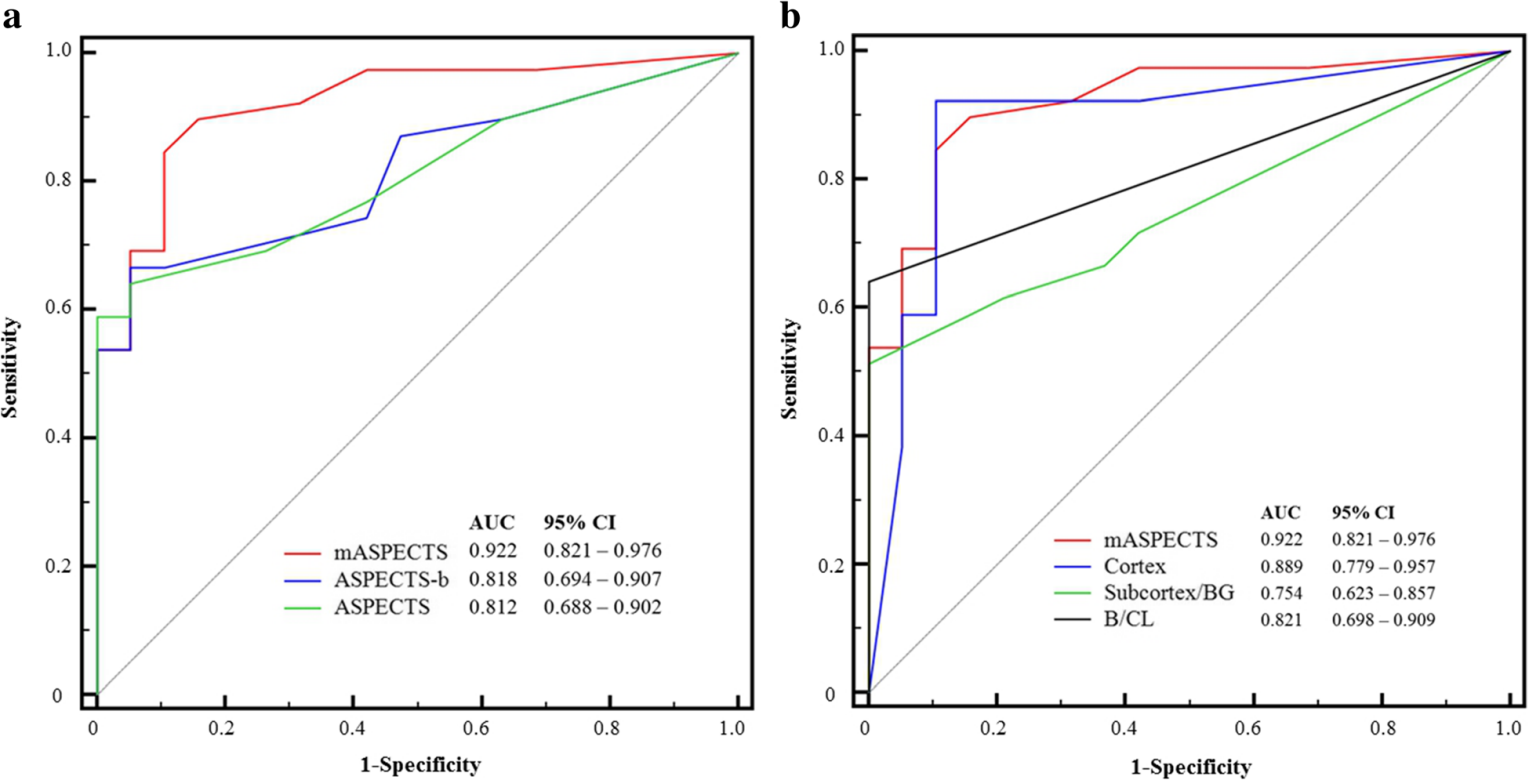
ROC curves for the prediction of poor neurological outcome using ASPECTS, ASPECTS-b, and mASPECTS (**a**), as well as three regions of mASPECTS (**b**). *ASPECTS* Alberta Stroke Program Early Computed Tomography Score, *ASPECTS-b* Bilateral ASPECTS, *mASPECTS* Modified ASPECTS, *BG* Basal ganglia, *B* Brainstem, *CL* Cerebellum

In this study, interrater reliabilities of CT scores were excellent. ICCs of ASPECTS, ASPECTS-b, and mASPECTS were 0.918 (95% CI, 0.865–0.950), 0.918 (95% CI, 0.866–0.951), and 0.915 (95% CI, 0.860–0.949), respectively. CT scores of ASPECTS, ASPECTS-b, and mASPECTS in the poor neurological outcome group were significantly lower than those in the good neurological outcome group (all *p* < 0.001) (Table 4, Fig. 3a). In ROC curve analysis for the prediction of poor neurological outcome, the C-statistic of ASPECTS was 0.812 (95% CI, 0.688–0.902). A cutoff ≤ 3 had a sensitivity of 59.0% (95% CI, 42.1–74.4%) and a specificity of 100% (95% CI, 82.4–100%). The C-statistic of ASPECTS-b was 0.818 (95% CI, 0.694–0.907). A cutoff ≤ 14 had a sensitivity of 66.7% (95% CI, 49.8–80.9%) and a specificity of 94.7% (95% CI, 74.0–99.9%). The C-statistic of mASPECTS was 0.922 (95% CI, 0.821–0.976). A cutoff ≤ 25 had a sensitivity of 84.6% (95% CI, 69.5–94.1%) and a specificity of 89.5% (95% CI, 66.9–98.7%). The predictive performance of mASPECTS for poor neurological outcome was better than that of ASPECTS or ASPECTS-b (*p* = 0.004 and *p* = 0.003, respectively). Among three regions (cortex, subcortex/basal ganglia, and brainstem/cerebellum) for mASPECTS, the score of cortex had the best predictive performance for poor neurological outcome after ECPR (Fig. 3b).

**Table 4:** mASPECTS and ASPECTS-b according to neurological outcomes

	Good neurological outcome (*n* = 19)	Poor neurological outcome (*n* = 39)	*p* Value
ASPECTS (range 0–10)	9.0 (7.5–10.0)	1.0 (0–8.0)	< 0.001
ASPECTS-b^a^ (range 0–20)	19.0 (16.0–20.0)	6.0 (0–17.5)	< 0.001
mASPECTS^b^ (range 0–31)	29.0 (27.0–31.0)	9.0 (0–25.0)	< 0.001
Cortex (range 0–20)	20.0 (18.5–20.0)	4.0 (0–14.0)	< 0.001
Subcortex/basal ganglia (range 0–8)	8.0 (6.0–8.0)	0 (0–8.0)	0.001
Brainstem/cerebellum (range 0–3)	3.0 (3.0–3.0)	0 (0–3.0)	< 0.001

*ASPECTS* Alberta Stroke Program Early Computed Tomography Score

^a^ASPECTS-b; ASPECTS-b is extended to both sides from the original ASPECTS protocol

^b^mASPECTS, modified ASPECT; similar to ASPECTS-b (bilateral ASPECTS), each middle cerebral artery territory had 0–10 points. Anterior cerebral artery and posterior cerebral artery territories had 2 points, corresponding to upper and lower levels of each territory. The brainstem and each cerebellar hemisphere had 1 point

There were only ten patients for whom follow-up CT scans were performed within 7 days (three good neurological outcomes and seven poor neurological outcomes) during ECMO support. Four patients had lower CT scores than previous scans, and another six patients had the same scores as for previous scans. All four patients who had lower CT scores than on previous scans had poor neurological outcomes.

Portable EEG was performed in 30 patients within 7 days after ECPR. Continuous slow (36.7%) and background suppression (23.3%) were the most common EEG findings. One-half of patients received continuous intravenous sedatives during EEG monitoring. Therefore, EEG findings might have reflected the use of sedatives as well as hypoxic brain injury. All patients who had well-known malignant EEG patterns such as electrocerebral inactivity, burst suppression, or generalized periodic epileptiform discharges had poor neurological outcomes. The EEG findings are shown in Additional file 1.

## Discussion

In the present study, we evaluated whether ASPECTS with some modifications could be used to predict neurological outcomes in patients after ECPR. Major findings of this study are as follows:

1. Brain CT could be helpful for predicting neurological outcomes of post-cardiac arrest patients after ECPR.
2. ASPECTS and its modifications were feasible and reliable for predicting neurological outcomes of post-cardiac arrest patients after ECPR.
3. The predictive performance of mASPECTS for poor neurological outcome was better than that of ASPECTS or ASPECTS-b.
4. In multivariable analysis, ischemic cardiac cause of arrest and mASPECTS were significant predictors of poor neurologic outcomes in ECPR patients.

Neuroimaging studies such as brain CT, brain magnetic resonance imaging, and magnetic resonance spectroscopy after conventional CPR have been performed [1]. Brain CT scans might be particularly helpful for predicting neurological outcomes after ECPR. However, brain magnetic resonance imaging is an expensive and time-consuming procedure. It cannot be performed immediately, because ECMO equipment is used for patients receiving ECPR [22]. In addition, sedative medications and neuromuscular blockade are commonly used in comatose CPR survivors. They might confound outcome prediction after cardiac arrest [23]. Some clinical signs and examinations such as motor response to noxious stimuli, corneal reflex, caloric testing, and EEG might also be confounded by sedation [1, 23]. Therefore, brain CT might be more helpful for predicting neurological outcome in ECPR patients under sedation. However, data on prognostic markers of brain CT for neurological outcomes after ECPR are limited [17, 24].

Some changes visualized by brain CT have been associated with hypoxic-ischemic cerebral insult. They are useful for predicting poor neurological outcome after cardiac arrest [5–7, 25]. Especially, loss of the boundary between gray matter and white matter and cortical sulcal effacement on brain CT are associated with hypoxic-ischemic brain damage and poor outcome after cardiac arrest [5–7, 25]. These changes are also useful for predicting poor neurological outcomes after ECPR [7]. However, identification of these signs might depend on the abilities of each investigator. In addition, these changes are not quantifiable. Therefore, an objective and quantifiable tool is needed to identify hypoxic-ischemic lesions. ASPECTS is a widely used screening tool to quantify the extent of ischemic hypodensity or hypoattenuation in ischemic stroke. ASPECTS and its modifications might be able to explain the quantitative association between cerebral insult following hypoxic-ischemic injury after cardiac arrest and neurological outcome [9, 10]. In conventional CPR, ASPECT with some modifications could be used to estimate early neurologic outcomes in post-cardiac arrest patients [6, 9, 10].

Selective vulnerability has been reported for hypoxic-ischemic injury in previous studies. The middle lamina of the cortex, caudate nucleus, putamen, globus pallidus, thalami, and Purkinje cells might be at particular risk owing to highly metabolically active tissue [26, 27]. Eventually, cortex, basal ganglia, and the cerebellum are areas vulnerable to hypoxic-ischemic injury after cardiac arrest. In the present study, the performance of mASPECTS for predicting poor neurological outcome was better than ASPECTS-b. Although ASPECTS-b can estimate hypoxic-ischemic lesions in bilateral MCA regions (MCA territorial cortex, subcortex, and basal ganglia), mASPECTS can estimate lesions in bilateral regions of ACA, PCA, and the cerebellum as well as MCA regions of ASPECTS-b. Therefore, mASPECTS can better detect cortical lesions by hypoxic-ischemic insult than ASPECTS-b. In addition, cortical lesions seem to be more relevant to neurological outcomes after ECPR than those of subcortex/basal ganglia or the brainstem/cerebellum. These findings suggest that mASPECTS is more useful than ASPECTS or ASPECT-b for predicting neurological outcomes in patients after ECPR.

This study has several limitations. First, the CPC score was retrospectively determined on the basis of medical records. Second, the nonrandomized nature of the registry data might have resulted in selection bias. Although brain CT was performed within 48 hours following ECPR, a major limitation of this study might be that the CT was performed at different time settings. However, in ROC curves for the prediction of poor neurological outcome using CT scores, AUCs of CT scores were not significantly different between within and after median CT scan intervals in this study. Third, brain CT scan was performed by two different scanners, which might have resulted in bias. Fourth, selection bias might have been an influence because we excluded 26 non-survived patients for whom we could not exactly define neurological status at discharge. We added the result including these 26 patients to compare with the results excluding these patients in Additional file 2. Last, a limited number of patients received targeted temperature management. The methods used were decided by each intensivist. They varied according to conditions and situations.

## Conclusions

In this study, ASPECTS with modifications was found to be feasible and reliable for predicting neurological outcomes in ECPR survivors.

## Key messages

- Brain CT can be helpful for predicting neurological outcomes of post-cardiac arrest patients after ECPR.
- ASPECTS and its modifications were feasible and reliable for prediction of neurological outcomes of post-cardiac arrest patients after ECPR.
- The predictive performance of mASPECTS for poor neurological outcome was better than that of ASPECTS or bASPECTS.

## Additional files

Additional file 1: **Table S1.** The findings of portable electroencephalography (EEG) within 7 days after extracorporeal cardiopulmonary resuscitation. (DOC 29 kb)

Additional file 2: **Figure S1.** mASPECTS according to neurological outcome. Bar is presented to median with interquartile range. There was a significant difference in mASPECTS according to neurological outcome (*p* < 0.001), and it was assessed by the Kruskal-Wallis test among groups. Bars of the same color indicate nonsignificant differences between groups based on the Mann-Whitney *U* test. (DOCX 172 kb)

## Abbreviations

ACA: Anterior cerebral artery
ASPECTS: Alberta Stroke Program Early Computed Tomography Score
ASPECTS-b: Bilateral ASPECTS
CPC: Cerebral Performance Categories scale
CPR: Cardiopulmonary resuscitation
CT: Computed tomography
ECMO: Extracorporeal membrane oxygenation
ECPR: Extracorporeal cardiopulmonary resuscitation
EEG: Electroencephalography
HUMC: Hallym University Medical Center
ICC: Intraclass correlation coefficient
ICU: Intensive care unit
mASPECTS: Modified ASPECTS
MCA: Middle cerebral artery
PCA: Posterior cerebral artery
ROSC: Return of spontaneous circulation
SMC: Samsung Medical Center
