# Supplementary material for: Prognostic value of computed tomography score in patients after extracorporeal cardiopulmonary resuscitation
Source: Crit Care. 2018 Nov 22;22:323. doi: 10.1186/s13054-018-2101-2 (PMC6251141; doi:10.1186/s13054-018-2101-2)
Supplement: Supplementary file 1 — Table S1. The findings of portable electroencephalography (EEG) within 7 days after extracorporeal cardiopulmonary resuscitation. (DOC 29 kb) [file 13054_2018_2101_MOESM1_ESM.doc]

**Supplementary table 1.** The findings of portable electroencephalography (EEG) within 7 days after extracorporeal cardiopulmonary resuscitation.

|  | Good neurological outcome (n = 11) | Poor neurological outcome (n = 19) | *p* value |
| --- | --- | --- | --- |
| EEG findings — no. of patients (%)  Continuous slow  Background suppression  Electrocerebral inactivity  Burst suppression  Generalized periodic epileptiform discharges  Ictal EEG | 9 (81.8)  2 (18.2)  0 (0)  0 (0)  0 (0)  0 (0) | 2 (10.5)  5 (26.3)  8 (42.1)  1 (5.3)  2 (10.5)  1 (5.3) | 0.005 |
