# Supplementary material for: Prognostic value of computed tomography score in patients after extracorporeal cardiopulmonary resuscitation
Source: Crit Care. 2018 Nov 22;22:323. doi: 10.1186/s13054-018-2101-2 (PMC6251141; doi:10.1186/s13054-018-2101-2)
Supplement: Supplementary file 2 — Figure S1. mASPECTS according to neurological outcome. Bar is presented to median with interquartile range. There was a significant difference in mASPECTS according to neurological outcome (p < 0.001), and it was assessed by the Kruskal-Wallis test among groups. Bars of the same color indicate nonsignificant differences between groups based on the Mann-Whitney U test. (DOCX 172 kb) [file 13054_2018_2101_MOESM2_ESM.docx]

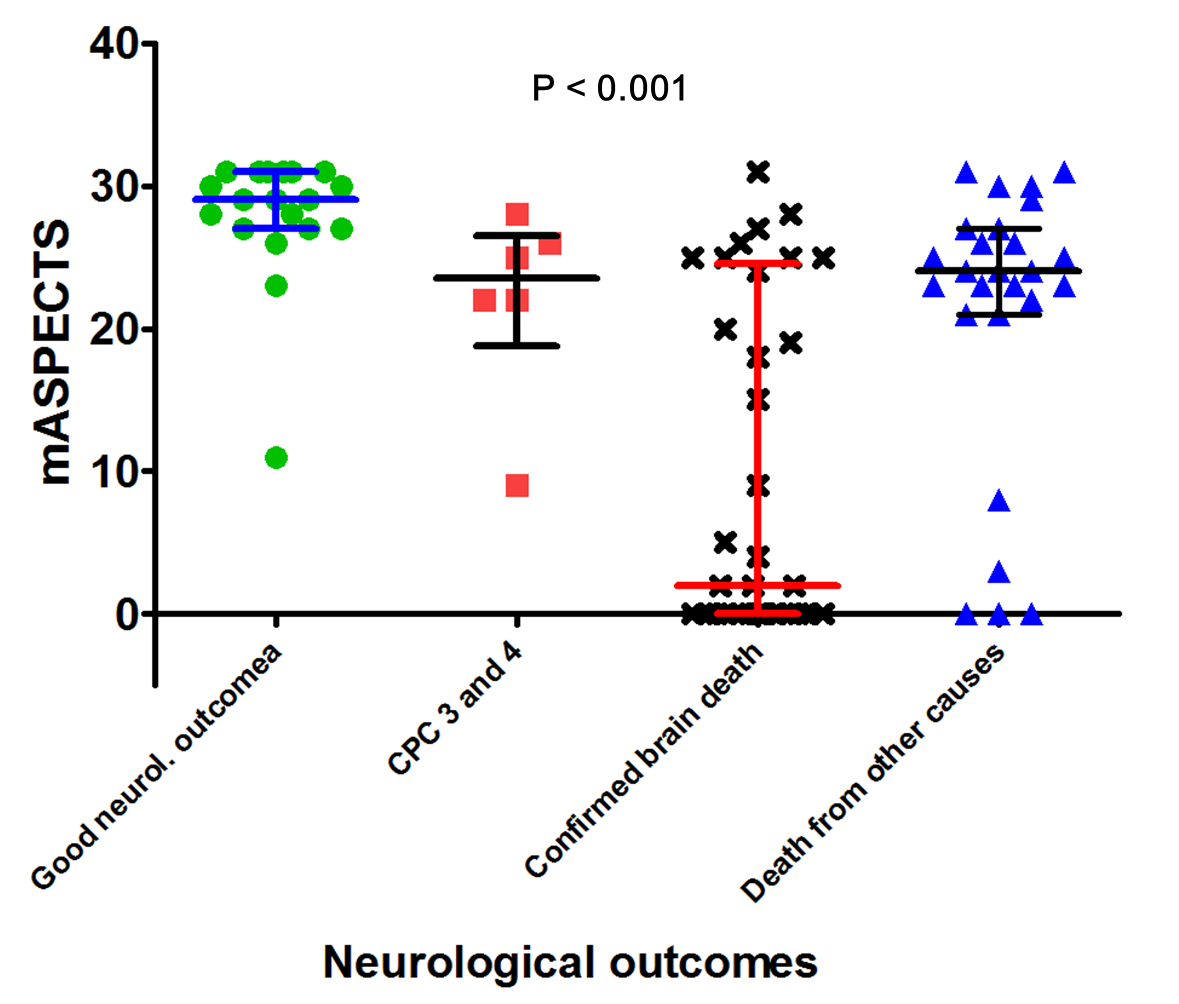


**Supplementary figure 1.** mASPECTS according to neurological outcome. Bar is presented to median with interquartile range. There was significant difference in mASPECTS according to neurological outcome (*p* < 0.001) and it was tested by the Kruskal-Wallis test among groups. Bars of the same color indicate non-significant differences between groups base on the Mann-Whitney U test. The patients of confirmed brain death had lowest mASPECT scores (2.0 [0 – 24.0]) among the neurological outcome groups. Especially, mASPECTSs of patients died of brain death were lower compared with those of patients die of other causes (2.0 [0 – 24.0] vs. 24.0 [21.0 – 27.0], *p* = 0.02). In addition, mASPECTSs were not significant difference between patients with CPC 3 – 4 and patients died of causes other than brain death (*p* = 0.884). mASPECTS, modified Alberta stroke program early computed tomography score; CPC, Cerebral Performance Categories.
